# A case of biliary adenofibroma of the liver with malignant transformation: a morphomolecular case report and review of the literature

**DOI:** 10.1186/s40792-019-0661-2

**Published:** 2019-06-24

**Authors:** Anne-Kathrin Sturm, Thilo Welsch, Christoph Meissner, Daniela E. Aust, Gustavo Baretton

**Affiliations:** 1 Institute for Pathology, University Hospital Carl Gustav Carus, TU Dresden, Fetscherstraße 74, 01307 Dresden, Germany; 2 Department of Visceral, Thoracic and Vascular Surgery, University Hospital Carl Gustav Carus, TU Dresden, Fetscherstraße 74, 01307 Dresden, Germany; 3 Department of Radiology, University Hospital Carl Gustav Carus, TU Dresden, Fetscherstraße 74, 01307 Dresden, Germany

**Keywords:** Biliary adenofibroma, Bile duct adenoma, Intrahepatic cholangiocarcinoma, Ductal plate malformation, Liver

## Abstract

**Background:**

Biliary adenofibroma is an exceptionally rare benign liver tumor with the potential for malignant transformation. In literature, only 21 cases have been described.

**Clinical presentation:**

In a healthy 63-year-old woman, a partly solid, partly cystic mass in the left lobe of the liver during a routine ultrasound examination was found. The computed tomography (CT) scan of the abdomen showed a 6.3 × 5.0-cm multilobulated cystic, partly hypervascularized mass in the liver segment IVa, with extension into segments II and IVb. There was no evidence of lymph node or distant metastases. Extirpation of the tumor was indicated by the multidisciplinary tumorboard. Microscopic examination showed a biphasic composed tumor with tubules embedded in fibrous stroma. In addition, there were also areas with pseudopapillary projections, as well as parts with focal cribriform-like growth pattern, which have been indicated as a possible sign of malignant transformation. Additionally, we found two different polymorphisms in the encoded TP53 und KIT in both distinct morphology tumor areas by molecular analysis, which ensured a tumor in malignant transformation. The patient has been alive for 24 months after R0 resection without tumor recurrence. Further investigation of more cases of this rare entity is necessary to proof molecular genesis.

**Conclusions:**

We report a rare case of a biliary adenofibroma with transition to an intrahepatic cholangiocellular carcinoma and present a brief literature review.

## Background

Biliary adenofibroma is a very rare liver tumor. According to the WHO 2010, biliary adenofibroma is classified as a benign tumor. On the other hand, several authors attributing malignant potential to this entity. Depending on tumor diameter, proliferation activity, and p53 accumulation, the lesions are considered to be premalignant neoplasias [1]. Histogenesis and biological behavior are still poorly understood. Herein, we report a morphomolecular case of a biliary adenofibroma with transition to an intrahepatic cholangiocellular carcinoma and present a brief literature review.

## Clinical presentation

**Fig. 1:**
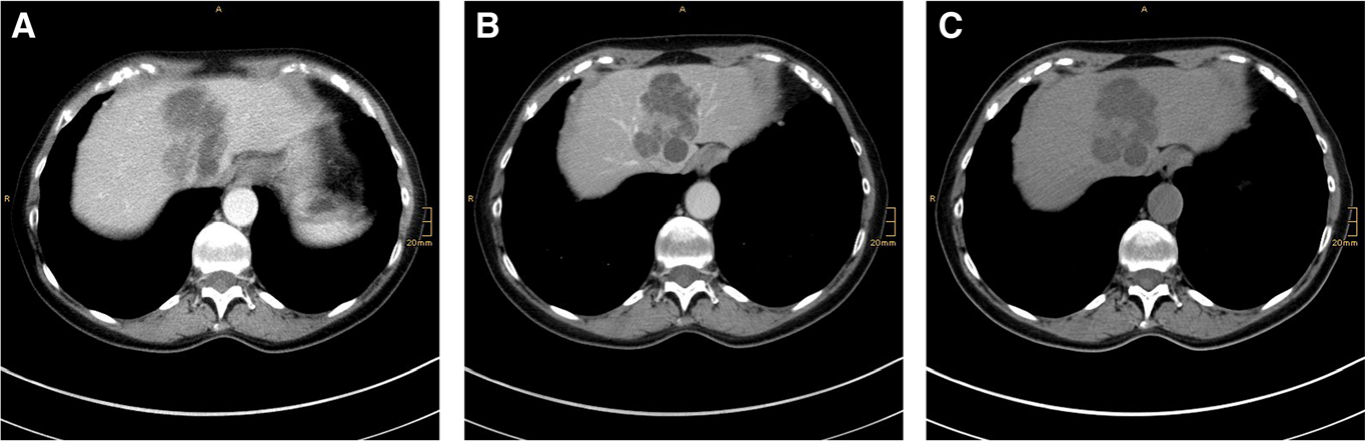
Radiologic findings. Axial CT image with contrast though the liver shows a cystic mass from segment IVa, with extension into segments II and IVb. **a** Hepatic arterial phase. **b** Portal venous phase. **c** Native

In a 63-year-old female patient, an ultrasound examination due to unspecific abdominal complaints showed a partly solid, partly cystic mass in the left lobe of the liver. The consecutive CT scan showed a 6.3 × 5-cm multilobulated, cystic, partly hypervascularized tumor in liver segment IVa, with extension into segments II and IVb (Fig. 1). The lesion displaced the left and middle hepatic veins and extended cranially just below the liver capsule. It has a malignant appearance with arterial and portal venous phase venous enhancement, but not typically for one of the common liver tumors like CCC, HCC, or metastasis. There was no evidence for the presence of hepatic metastasis or a primary tumor located elsewhere in diagnostic imaging. MRI which would be the gold standard was not performed.

The patient had no other concomitant disease. Notably, inquiring family history, the grandmother and father had died of “liver cancer”. Alcohol and nicotine consumption was denied. Exposure to liver-toxic substances could not be determined. The physical examination and the laboratory revealed no pathological findings. The tumor markers including AFP, CEA, and CA 19-9 were not elevated.

In sonographically guided transcutaneous biopsy of the liver, the lesion was histomorphologically rich in connective tissue with irregularly shaped and in part significantly dilated glandular structures with mild atypia and increased hyperchromasia of the cells. Immunohistochemistry showed expression of CK7 in the absence of immune reaction with antibodies to cadherin 17, TTF-1, and S100P. The finding was considered consistent with a primary adenocarcinoma of the bile duct in accordance with the radiological imaging. Based on these findings, in our interdisciplinary tumor board, decision was made for surgical resection. A left hemihepatectomy was performed and the postoperative course was uneventful. Postoperative follow-up and computed tomography 24 months postoperatively showed no evidence of recurrence or metastasis.

## Pathological findings

**Fig. 2:**
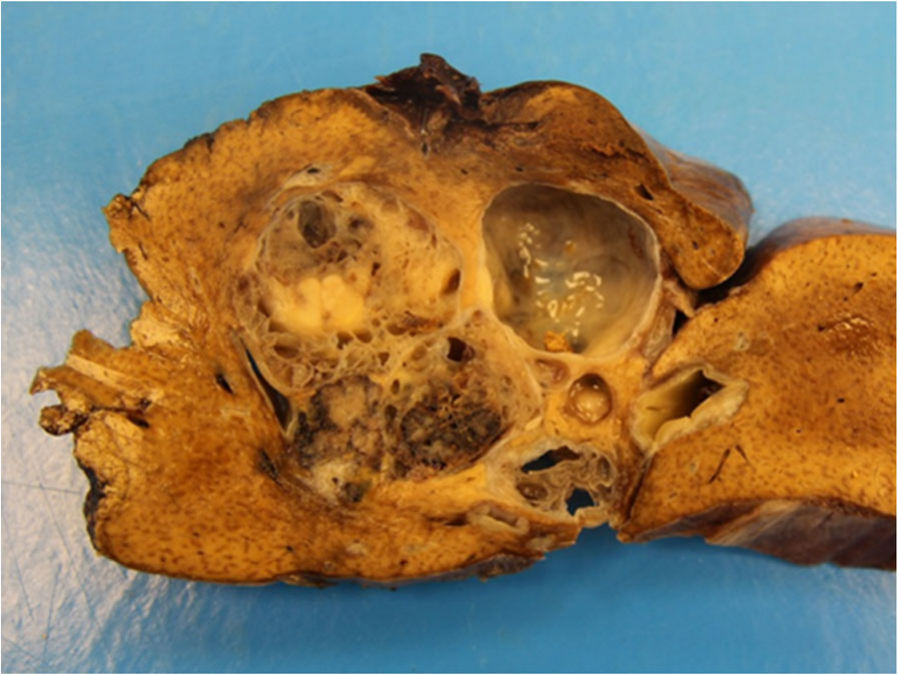
Gross findings. The cut surface shows numerous variable- sized cystic space admixed with solid areas

The left hemihepatectomy specimen (14.5 × 14.5 × 5.9 cm) showed macroscopically a well-defined, micro- to small-cystic lesion with focal solid areas, a maximum of 65 mm in diameter, and a gray cut surface (Fig. 2). The cysts discharged a clear, non-mucinous fluid. The remaining liver parenchyma was homogeneously grayish brown on the cut surface.

**Fig. 3:**
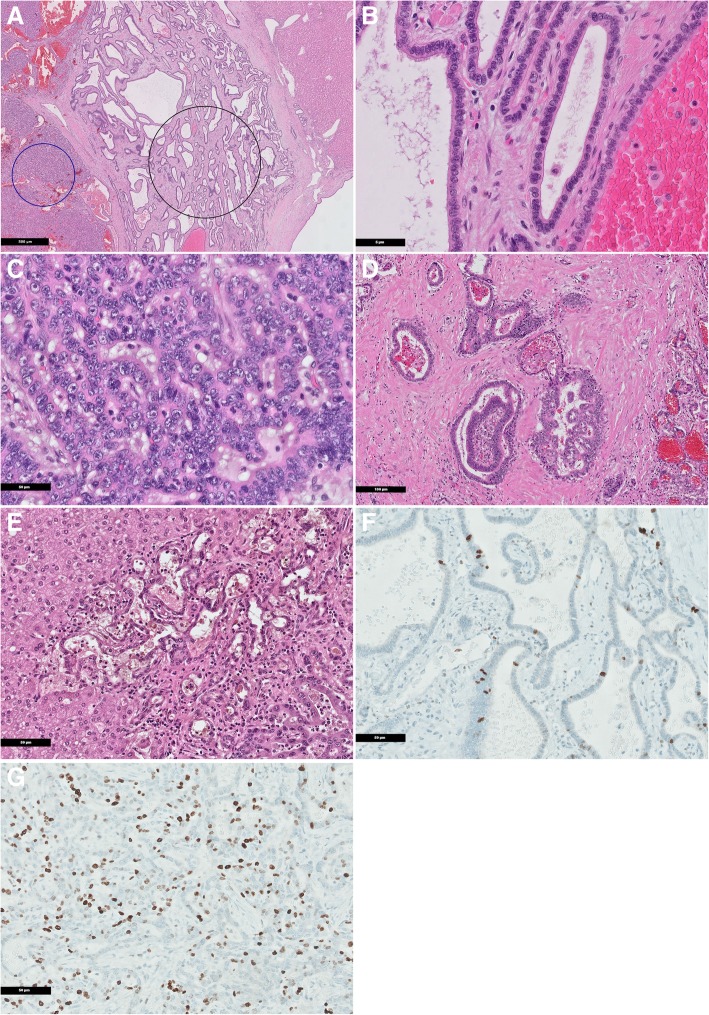
**a** Microscopic findings of the tumor. Low magnification shows crowded, back-to-back tubulare structures (left side; adenocarcinoma) and parts of tubulocystic formations embedded in dense fibrous stroma (center; biliary adenofibroma) with adjacent normal liver parenchym (right side) (H&E × 20). The black circle indicates the bengin part of tumor (bililary adenofibroma) and the blue circle the malignant part of tumor (adenocarcinoma). These two different parts of tumor were marked on the histological slide for subsequent dissection. **b** At higher magnification, the cysts lined by a bland cuboidal single layer to columnar epithelium of the biliary phenotype with uniform nuclei without feature of malignancy; biliary adenofibroma (H&E × 400). **c** The areas of macroscopically solid appearance shows irregular pattern with crowded, anastomosing tubules. The nuclei closely packed, pseudostratified with heterogeneously distributed chromatin and prominent nucleoli; adenocarcinoma (H&E × 400). **d** Focal, the neoplasm shows a complex architecture, composed of pseudopapillary and cribriform-like tubules in a dense stroma; adenocarcinoma (H&E × 100). **e** In the peripheral area reveals irregularly glandular structures infiltrate the adjacent liver parenchyma; adenocarcinoma (H&E × 200). **f** Ki67 proliferation index in benign part of the tumor (biliary adenofibroma) less than 10 % (Ki67 staining × 200). **g** Ki67 proliferation index in the malignant part of the tumor (adenocarcinoma) significantly increased to 20–30 % (Ki67 staining × 200)

Histologically, the tumor was composed of two distinct areas. The majority of the lesion showed multiple ducts variable in size, irregular in shape, and angulated with dilation to cysts, lined by a cuboidal single layer to columnar epithelium of the biliary phenotype. Mucin or bile production was not apparent. The tumor was in parts sharply demarcated with a pseudocapsular fibrosis facing the adjacent liver parenchyma (Fig. 3a). The tumor cells showed relatively monomorphic round to oval nuclei with a predominantly fine chromatin and in part with small, inconspicuous nucleoli and an eosinophilic cytoplasm (Fig. 3b). Mitoses were rarely found here (< 1 per 10 high-power fields (HPF)). The cell borders were well defined. In the lumen of tubulocystic proliferations were varying degrees of erythrocytes most likely due to secondary bleeding. The multicystic lesion was embedded in a dense and partially hyalinized, variable broad fibrous spindled stroma. The stroma showed no ovarian-like stromal changes. In the stroma, there was a variably densely mixed inflammatory infiltrate. The other part of the tumor showed crowded, back-to-back, anastomosing tubules with closely packed nuclei. In these areas, a more columnar type arrangement with predominantly basal oriented and partly pseudostratified nuclei was seen. The nuclei were characterized by low to moderate atypia. The nuclear membrane showed distinct contour and predominantly prominent nucleoli. The mitotic rate was here very rare (< 1 per 10 HPF). Atypical mitotic figures could not be detected. In this part, the tumor was accompanied by a tender, sometimes hardly distinguishable stroma (Fig. 3c). In addition, there were also areas with pseudopapillary projections, as well as parts with a focal cribriform-like growth pattern (Fig. 3d). In these areas, invasive growth in the adjacent liver parenchyma could be detected (Fig. 3e). There was no lymphangiosis or hemangiosis carcinomatosa.

Immunohistochemically, the epithelial cells stained diffusely positive for cytokeratin 7 and focally positive in single cells/cell groups for Cadherin 17 and CD56. Individual cell nuclei showed weak nuclear staining for p53. Muc1 was expressed on the apical surfaces of most epithelial cells. The stroma cells were diffusely smooth muscle-actin positive. The markers inhibin, calretinin, S100P, ERG, Muc2, Muc4, Muc5, and Muc6 were completely negative. Immunohistochemistry of the mismatch repair proteins revealed nuclear expression obtained from the markers MLH1, MSH2, MSH6, and PMS2. The Ki67 labeling index differs in the benign and malignant tumor components, from 5–10 % in biliary adenofibroma to 20–30% in the adenocarcinoma (Fig. 3f, g).

In addition, parts of the biliary adenofibroma as well as the cholangiocellular carcinoma component were examined for mutational status. The material was macrodissected and purified DNA was sequenced with Next-Generation Sequencing on the MiSeq platform (Illumina). The following genes were examined by the target enrichment using the TruSight Tumor Panel 15: AKT 1, BRAF, EGFR, ERBB2, FOXL2, GNA11, GNAQ, KIT, KRAS, MET, NRAS, PDGFRA, PIK3CA, RET, and TP53. No pathogenic variants could be found in the mentioned gene sections. Only two different polymorphisms (NM_000546.5: c.215C> G, TP53; NM_000222.2: c.1621A> C, KIT) were found in the biliary adenofibroma well as in the cholangiocellular carcinoma.

## Discussion

This case report describes one of the very rare cases of a biliary adenofibroma of the liver with proven transition to a well-differentiated adenocarcinoma of the biliary type.

Tsui et al. [2] were the first to describe this cystic liver lesion as a benign, complex, tubulocystic tumor with a bland spindle-cell stromal component. To date, there are only 21 case reports in the literature, 7 of which showed malignant transformation in a pre-existing biliary adenofibroma (Table 1). In majority, the reported biliary adenofibromas showed transition into papillary or tubulopapillary growth patterns. This histomorphological picture suggests a neoplastic progression of the tumor. Kaminsky et al. also described a microcystic growth pattern, as well as a neuroendocrine differentiation [3]. Another case showed pulmonary metastases after 3 years [4].

**Table 1:** Reported biliary adenofibromas of the liver (*n* = 22)

No.	Reference	Age/sex	Tumor size (cm)	p53	Ki67 (%)	Evidence of malignant transformation	Mutations found
1	Tsui et al. [2]	74/f	7			No	
2	Parada et al. [5]	49/f	7			No	
3	Akin and Coskun [4]					Pulmonary metastasis	
4	Garduno-Lòpez et al. [6]	68/m	6			No	
5	Varnholt et al. [7]	21/f	16			No	
6	Gurrera et al. [8]	79/m	5.5			No	
7	Kai et al. [9]	40/m	7	Negative	5–10	Atypias	
8	Nguyen et al. [10]	53/f	6.5			Yes	
9	Nakanuma et al. [11]	69/f	3.5	Focally positive	10–15	Yes	
10	Jacobs et al. [12]	57/f	10			Atypias	
11	Thai et al. [13]	77/m	4.8			Yes	
12	Godambe et al. [14]	71/f	6.3	50%	20–50	Yes	
13	Thompson et al. [15]	71/m	14.5			Yes	Nonsense mutation in p16 INK4a
14	Kaminsky et al. [3]	37/f	4.5	Negative	50	Yes	
15	Arnason et al. [16]	83/m	7				
16		47/f	16		6	Atypias	Large region gains in chromosome 1q; loss of 1p, 2p, 3q, 6q, 8p, 11p, 12q, 14, 16q
17		57/f	10; 2.5; 1.7	Positive	< 10	Atypias	gain of 1q; loss of 11q, 22q, Xq; focal amplifications of CCND1 and ERBB2
18		70/f	12	Negative	< 8	Atypias	Gain of 1q, 4p, 5, 8, 12p; losses of 1p, 4q, 6q, 11p, 14q, 17p
19		74/f	7	Negative	2	No	No chromosomal changes
20		46/m	15	Patchy positive	< 1	Atypias	
21	Esteban et al. [17]	26/f	2.6			No	
22	Sturm et al. (2019) (present case)	63/f	6.5	Focally positive	20–30	Yes	TP53 and KIT (NM_000546.5: c.215C> G, TP53; NM_000222.2: c.1621A> C, KIT)

Currently, the WHO (2010) lists two cystic intrahepatic lesions: non-invasive mucinous cystic neoplasia with mild to severe dysplasia and mucinous cystic neoplasia with associated invasive carcinoma [1]. Mucinous cystic neoplasms in older nomenclatures were referred to as cystadenoma or cystadenocarcinoma. By definition, the tumors show a mucinous epithelium with papillary projections and an ovarian-like spindle cell stroma. The tumor presented here did not show any characteristic features of a mucinous cystic neoplasia, in particular, immunohistology was negative with antibodies to inhibin in the stroma, which is usually positive in mucinous neoplasias.

In differential diagnosis, a rarely reported primary hepatic cystic mesothelioma was excluded by the lack of immune response to calretinin antibody [18, 19].

In addition, a biliary adenofibroma is morphologically reminiscent of a von Meyenburg complex. These benign hepatic lesions are typically less than 0.5 cm in size and are assigned to the shape of the ductal plate malformation. Isolated cases with a transition to dysplasia or associated carcinoma have also been observed in these small subcapsular tubulocystic lesions [20].

Similar to the cases described in the literature (Table 1), the presented neoplasia morphologically showed a characteristic structure of a biliary adenofibroma with tubulocystic biliary epithelium and a broad fibrous, non-ovarian-like stroma. In addition, a partly abrupt, partly gradual transformation into more cell-dense tumor areas could be seen, which histologically showed characteristics of an invasive carcinoma in the form of microcystic, fine-papillary and partly cribriform epithelial formations with distinct nuclear atypia and an increased mitotic rate, comparable to the two cases described by Thompson et al. with a very similar architecture [15].

Moreover, the biliary adenofibroma and the cholangiocellular carcinoma were examined with molecular pathology analysis. The two polymorphisms found in the encoded TP53 and KIT (NM_000546.5: c.215C> G, TP53; NM_000222.2: c.1621A> C, KIT) imply that the carcinoma developed from the biliary adenofibroma since it is not a “true” mutation in the sequence-specific DNA-binding domain of the TP53 tumor suppressor gene between amino acids 102–292 [21]. Within this sequence, the most common mutations that would result in a missense or nonsense mutation are located.

Arnason et al. reported six cases with biliary adenofibroma without histological features in transition into a malignant tumor. Three tumors of them tested by array comparative genomic hybridization showed chromosomal copy number alterations, including one with amplification of CCND1 and ERBB2 [16]. These amplifications, which alter cell cycle progression, are observed frequently in a variety of tumors and may contribute to tumorigenesis.

Some authors have investigated p53 immunohistochemistry staining to predict biological behavior. However, in immohistochemistry, the p53 positivity varies widely in the literature between only single positive cell nuclei up to 50% of the total tumor [14]. Molecular pathology studies of the biliary adenofibroma have not been extensively described. Only Thompson et al. found a nonsense mutation in the tumor suppressor protein p16 ^INK4a^, which encodes the cyclin-dependent kinase inhibitor (CDKN2A) in the gene [15].

## Conclusions

We present the rare case of a biliary adenofibroma showing transition into a well-differentiated intrahepatic cholangiocarcinoma. The malignancy was subsequently proven on the basis of histomorphological criteria, like an invasive growth in the adjacent liver parenchyma, crowed growth with depletion of the stoma, prominent nucleoli, nucleus-plasma ratio, and desmoplastic stroma reaction. This tumor entity is poorly understood. More cases are required for the accurate characterization of this rare entity and its premalignant properties.

## Data Availability

None.

## Abbreviations

AFP: Alpha-fetoprotein
Ca 19-9: Carbohydrate-Antigen 19-9
CEA: Carcinoembryonic antigen
CT: Computed tomography
DNA: Deoxyribonucleic acid
HPF: High-power field
MLH1: MutL homolog
MRI: Magnetic resonance imaging
MSH2: MutS protein homolog 2
MSH6: MutS protein homolog 6
Muc1: Mucin 1
Muc2: Mucin 2
Muc4: Mucin 4
Muc6: Mucin 6
PMS2: PMS1 Homolog 2-Mismatch Repair System Component
S100P: S100 calcium-binding protein P
WHO: World Health Organization
